# Renal Replacement Lipomatosis Presenting in the Setting of Ureteral Stricture with Absence of Renal Calculus Disease

**DOI:** 10.1155/2021/3640167

**Published:** 2021-10-23

**Authors:** Katrina Collins, Eric Brocken, Laura M. Warmke, Temel Tirkes, Michael Hwang

**Affiliations:** ^1^Department of Pathology, Indiana University, Indianapolis, IN 46202, USA; ^2^Department of Radiology and Clinical Sciences, Indiana University, Indianapolis, IN 46202, USA

## Abstract

Renal replacement lipomatosis of the kidney is a rare, benign entity in which extensive fibrofatty proliferation of the renal sinus is associated with marked atrophy of the renal parenchyma. It is often associated with calculi or long-standing inflammation. This entity may be confused with a fatty neoplasm of the kidney. A 51-year-old woman with a past medical history of pancreas transplant for type 1 diabetes subsequently developed ureteral stricture. This was initially managed by a nephrostomy tube and nephroureterostomy stenting with periodic exchanges to help restore urine flow; however, the renal function of the kidney progressively declined with recurrent and complicated urinary tract infections. She presented for kidney transplant with right native nephrectomy. Gross examination of the right kidney revealed a 12.8 cm renal sinus lipomatous mass replacing much of the kidney. Microscopically, the mass consisted of mature adipose tissue with fibrous septae and occasional thick-walled vessels with prominent smooth muscle bundles. A rare atypical stromal cell was present, otherwise no significant cytologic atypia or lipoblasts were identified. After excluding fat-predominant angiomyolipoma and well-differentiated liposarcoma, a diagnosis of renal replacement lipomatosis was made. Renal replacement lipomatosis is a benign condition typically associated with a nonfunctioning or poorly functioning kidney often linked to renal calculus disease or chronic renal infection. The presentation in our case was atypical given an absence of associated renal calculus disease. This case is intended to increase awareness of this less commonly encountered entity as it may be confused with a fatty neoplasm of the kidney, some with malignant potential.

## 1. Introduction

Renal replacement lipomatosis of the kidney is a rare, benign condition characterized by marked atrophy of renal parenchyma with fibrofatty proliferation of the renal sinus and is often associated with long-standing inflammation [1] and renal calculi in 76-79% of cases [2, 3]. Rare cases are idiopathic. In our report, we present a case of renal replacement lipomatosis not associated with renal calculus disease. The differential diagnosis and key imaging findings that served to establish this specific diagnosis with histopathologic confirmation are reviewed.

## 2. Case Presentation

**Figure 1:**
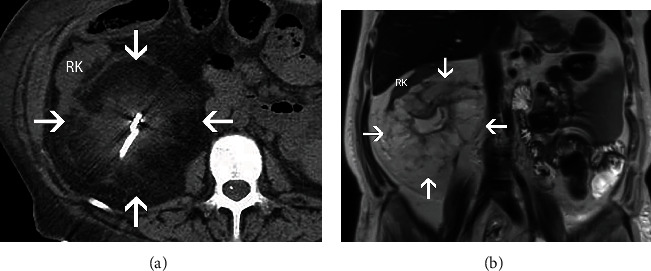
Imaging studies of the right kidney. (a) CT of the abdomen without contrast, axial view. At the level of the mass (outlined by white arrows), the right kidney (RK) is atrophic and there is extensive lipomatous proliferation of the renal sinus fat. The bright linear structure in the middle of the mass is a percutaneous drainage tube. (b) MRI of the abdomen without fat suppression or contrast, coronal T2- weighted image. There is a prominent hyperintense fatty proliferation of the renal sinus fat (outlined by white arrows) displacing the right kidney (RK). There is an associated thin rim of enhancing adjacent renal parenchyma.

**Figure 2:**
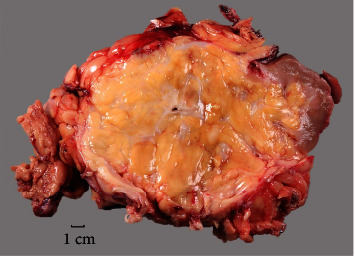
Gross photograph of bisected right kidney. The kidney is largely replaced by an extensive fatty proliferation of the renal sinus fat. There is adjacent surrounding residual compressed red-brown renal parenchyma.

**Figure 3:**
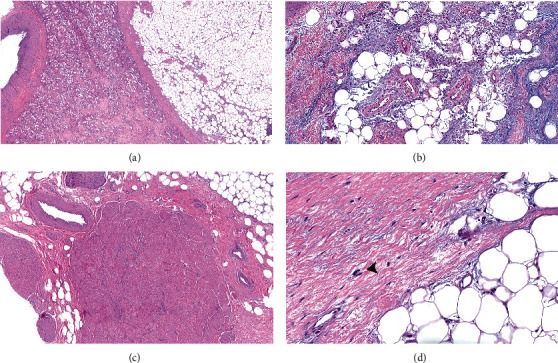
Microphotograph of the right kidney. (a) Well-demarcated mature adipose tissue and adjacent renal parenchyma. (b) Renal sinus fat showing inflammatory changes with areas of hyalinization and fat necrosis. (c) The lipomatous proliferation of the renal sinus fat contains fibrous septae and occasional thick-walled vessels with prominent smooth muscle bundles. (d) Rare atypical stromal cell (arrowhead), otherwise no significant cytologic atypia or lipoblasts were identified.

A 51-year-old woman with a past medical history of pancreas transplant for type 1 diabetes subsequently developed ureteral stricture. This was initially managed by a nephrostomy tube and nephroureterostomy stenting with periodic exchanges every two months to help restore urine flow; however, the renal function of the kidney progressively declined with recurrent and complicated urinary tract infections. She presented for kidney transplant with right native nephrectomy. Additionally, the patient has a history of left native nephrectomy at age 2 but the reason was not clearly documented. There was no previous history of renal calculi. Preoperative abdominal CT and MRI showed an atrophic right kidney with extensive lipomatous proliferation of the renal sinus fat (Figures 1(a) and 1(b)). There was extensive inflammation within the collecting system and a percutaneous drainage tube was present. On gross examination, a markedly distorted nephrectomy specimen was received with a moderate amount of attached perinephric fat, with a weight of 1,040 grams and overall dimensions of 19.7 × 12.7 × 10.4 cm. The cut surface revealed a 14.7 × 12.8 × 12.2 cm gray-yellow to white, lobulated, renal sinus lipomatous mass with fibrous septae replacing approximately 85% of the kidney (Figure 2). Areas of hemorrhage and degeneration were also noted. The residual renal parenchyma was red-brown and unremarkable. Microscopic examination revealed the mass consisted of mature adipose tissue with fibrous septae and occasional thick-walled vessels with prominent smooth muscle bundles. A rare atypical stromal cell was present, otherwise no significant cytologic atypia or lipoblasts were identified. There was a clear demarcation between adipose tissue and residual renal parenchyma at the periphery (Figures 3(a)–3(d)), which showed relatively mild chronic changes. Arteries showed fibrous intimal thickening. Mild patchy interstitial fibrosis and tubular atrophy were estimated to be less than 25%. Glomerular obsolescence was approximately 20%. Mild mesangial matrix expansion without well-developed Kimmelstiel-Wilson nodules by PAS stain was seen. Immunohistochemical studies with antibodies against HMB-45, Melan-A, and SMA did not support a diagnosis of renal angiomyolipoma. Immunohistochemistry and fluorescence *in situ* hybridization to detect MDM2 expression and *MDM2* amplification, respectively, were negative. After excluding a fat-predominant angiomyolipoma and well-differentiated liposarcoma, a diagnosis of renal lipomatosis was made. The patient remained asymptomatic after 8 months of follow-up.

## 3. Discussion

Renal replacement lipomatosis has been described using a variety of terms including renal fibrolipomatosis [4, 5], renal replacement lipomatosis of the kidney [2, 6, 7], fatty transformation of the kidney [8], fatty replacement of the kidney, lipomatous paranephritis, and lipoma diffusum renis [9]; however, the term renal replacement lipomatosis is most used at present. The first case was described in 1841 by Rayer [10] occurring in an autopsy performed in 1837 [11]. Several reported cases have been reported in the English literature, the majority of which are individual case reports and a few large case series [2, 3, 12]. Renal replacement lipomatosis is a rare, benign condition characterized by prominent fat proliferation of the renal sinus with atrophy of the renal parenchyma that may mimic other renal malignancies. It typically occurs unilaterally, but bilateral cases have been reported [13] and are thought to result in association with infection, obstructive renal calculi, or long-standing hydronephrosis. Rare cases are idiopathic [13–15]. A major differential diagnosis for infection, often associated with chronic obstruction from calculi, is xanthogranulomatous pyelonephritis (XGP). XGP is characterized by destruction of the renal parenchyma and replacement by lipid-laden macrophages, whereas renal lipomatosis is a fatty proliferation of the renal sinus with atrophy of the renal parenchyma [16–18]. Cases of XGP coexisting with renal lipomatosis have been reported [19–22] as well as with malignancy [23].

Many of the tumors of the renal pelvis include urothelial carcinoma, with squamous cell carcinoma accounting for a smaller percentage of these cases. Renal parenchymal tumors such as renal cell carcinoma commonly extend into the renal sinus. Rarely, tumors originating from mesenchymal tissue develop in the renal sinus. Renal lipomatosis may be confused with fat-containing renal tumors such as renal lipoma, angiomyolipoma, or liposarcoma. In contrast to renal lipomatosis, these tumors are generally located in intrarenal or extrarenal areas outside the renal sinus [24, 25]. Renal lipomas tend to be small and originate in the fat cells within the renal capsule. Renal liposarcomas arise peripherally in perirenal fat within Gerota's fascia or within the renal capsule and may distort the kidney but usually do not invade the adjacent renal parenchyma. Patients with lipoma, angiomyolipoma, or liposarcoma also tend to have no other associated pathology except in compressed areas immediately surrounding the tumor without impact on overall renal function [26], whereas with renal lipomatosis there is evidence of infection and displacement of normal renal structures by fat. Angiomyolipoma and liposarcoma can be easily differentiated from renal lipomatosis. The presence of smooth muscle fibers, thick-walled blood vessels, and coexpression of melanocytic and smooth muscle markers by immunohistochemistry in myoid and lipoid components in angiomyolipoma and atypical stromal cells with detection of MDM2 expression by immunohistochemistry and genetic amplification by fluorescence *in situ* hybridization is absent in renal lipomatosis.

In conclusion, renal lipomatosis may be confused with lipomatous neoplasms of the kidney. It is usually associated with a unilateral nonfunctioning or poorly functioning kidney. When present in a native kidney that is deemed nonfunctional, nephrectomy is often adequate treatment [17]. We want to raise awareness among pathologists of this entity, as it is infrequently encountered and may be difficult to differentiate from other fat-containing tumors in the renal sinus preoperatively.
